# The Clinicopathological Characteristics And Genetic Alterations of Signet-ring Cell Carcinoma in Gastric Cancer

**DOI:** 10.3390/cancers12082318

**Published:** 2020-08-17

**Authors:** Kuo-Hung Huang, Ming-Huang Chen, Wen-Liang Fang, Chien-Hsing Lin, Yee Chao, Su-Shun Lo, Anna Fen-Yau Li, Chew-Wun Wu, Yi-Ming Shyr

**Affiliations:** 1 Division of General Surgery, Department of Surgery, Taipei Veterans General Hospital, Taipei 11217, Taiwan; khhuang@vghtpe.gov.tw (K.-H.H.); chewwunwu@gmail.com (C.-W.W.); ymshyr@vghtpe.gov.tw (Y.-M.S.); 2 School of Medicine, National Yang-Ming University, Taipei 11221, Taiwan; ychao@vghtpe.gov.tw (Y.C.); sslo@ymuh.ym.edu.tw (S.-S.L.); fyli@vghtpe.gov.tw (A.F.-Y.L.); 3 Center of Immuno-Oncology, Department of Oncology, Taipei Veterans General Hospital, Taipei 11217, Taiwan; mhchen9@vghtpe.gov.tw; 4 Genome Research Center, National Yang-Ming University, Taipei 11221, Taiwan; jameslin@fcbiotech.com.tw; 5 Department of Surgery, National Yang-Ming University Hospital, Yilan 26058, Taiwan; 6 Department of Pathology, Taipei Veterans General Hospital, Taipei 11217, Taiwan

**Keywords:** signet-ring cell, genetic alterations, advanced GC, *PD-L1*, recurrence pattern, prognostic factor review, meta-analysis

## Abstract

Signet-ring cell carcinoma (SRC) in advanced gastric cancer (GC) is often associated with more invasiveness and a worse prognosis than other cell types. The genetic alterations associated with gastric carcinogenesis in SRC are still unclear. In this study, 441 GC patients receiving curative surgery for GC between 2005 and 2013 were enrolled. The clinicopathological characteristics and genetic alterations of GC patients with and without SRC were compared. Among the 441 GC patients, 181 had SRC. For early GC, patients with SRC had more tumors located in the middle and lower stomach, more infiltrating tumors and better overall survival (OS) rates than those without SRC. For advanced GC, patients with SRC had more scirrhous type tumors, more *PIK3CA* amplifications, fewer microsatellite instability-high (MSI-H) tumors, more peritoneal recurrences and worse 5-year OS rates than those without SRC. For advanced GC with SRC, patients with peritoneal recurrence tended to have *PD-L1* expression. For advanced GC without SRC, patients with liver metastasis tended to have *PD-L1* expression, *PI3K/AKT* pathway mutations, *TP53* mutations and MSI-H tumors. For advanced GC, *PD-L1* expression was associated with peritoneal recurrence in SRC tumors, while non-SRC tumors with liver metastasis were likely to have *PI3K/AKT* pathway mutations, *TP53* mutations and *PD-L1* expression; immunotherapy and targeted therapy may be beneficial for these patients.

## 1. Introduction

Gastric cancer (GC) is the sixth most common cancer and the second most common cause of cancer death worldwide [1]. Signet-ring cell carcinoma (SRC) is characterized by malignant tumor cells with prominent mucin in the cytoplasm and eccentric crescent-shaped nuclei. According to the classification of the World Health Organization (WHO) [2], SRC is defined as being present in more than 50% of GC tumors. Compared to other types of GC, SRC tends to occur predominantly in those who are younger and female and has a higher frequency of lymph node metastasis and a poor prognosis [3].

A meta-analysis study [4] demonstrated that for early GC (T1), SRC was associated with a better prognosis than non-SRC, while for advanced GC (T2-T4), SRC had a worse prognosis than non-SRC, which was similar to the findings of our previous report [5]. We hypothesize that the genetic alterations between SRC and non-SRC in early and advanced GC may be different, and it deserves investigating whether these genetic alterations are associated with patient survival. To date, the molecular difference between SRC and non-SRC in GC is still unclear; further investigation of the genetic alterations may provide useful information to explain the phenomenon.

The aim of the present study was to compare the clinicopathological characteristics and genetic alterations between patients with and without SRC in early and advanced GC. In addition, we investigated whether genetic alterations are associated with patient prognosis in SRC and non-SRC GC.

## 2. Results

### 2.1. Clinicopathological Features

Among the 441 patients, 181 had SRC. Among the 260 non-SRC patients, there were 5 papillary adenocarcinomas (1.9%), 240 tubular adenocarcinomas (92.3%) and 15 mucinous adenocarcinomas (5.8%), and no patient was diagnosed with undifferentiated adenocarcinoma.

We compared the clinicopathological characteristics between patients with and without SRC (Table 1). Univariate analysis showed that patients with SRC were younger and more likely to be female and had more tumors located in the middle stomach, more scirrhous stromal reaction, a more infiltrating type, fewer microsatellite instability-high (MSI-H) tumors, more *PIK3CA* amplifications, less *PD-L1* expression, fewer *PI3K/AKT* pathway mutations and more advanced Tumor, Node, Metastasis (TNM) stages than those without SRC. Multiple testing correction logistic regression demonstrated that patients with SRC were more likely to be female and had more scirrhous stromal reaction, a more infiltrating type, fewer MSI-H tumors and more *PIK3CA* amplifications than those without SRC.

With regard to early GC (Table 2), univariate analysis showed that patients with SRC were more likely to be female and had more tumors located in the middle stomach, a poorer differentiation and a more infiltrating type than those without SRC. Multiple testing correction logistic regression demonstrated that patients with SRC had more tumors located in the middle stomach and a more infiltrating type than those without SRC.

With regard to advanced GC (Table 3), patients with SRC were younger and more likely to be female and had more tumors located in the middle stomach, more scirrhous stromal reactions, a more infiltrating type, fewer MSI-H tumors, more *PIK3CA* amplifications, less *PD-L1* expression, fewer *ARID1A* mutations and more advanced TNM stages than those without SRC. Multiple testing correction logistic regression demonstrated that patients with SRC had more scirrhous stromal reactions, fewer MSI-H tumors and more *PIK3CA* amplifications than those without SRC.

With regard to the combined positive score (CPS) of *PD-L1* expression, there was no significant difference in CPS between patients with SRC and without SRC (0.49 ± 1.48 vs. 0.92 ± 2.56, *p* = 0.166), which was observed in both early GC (0.40 ± 1.13 vs. 0.43 ± 1.30, *p* = 0.915) and advanced GC (0.54 ± 1.65 vs. 1.09 ± 2.87, *p* = 0.199).

### 2.2. Initial Recurrence Patterns

As shown in Table 4, among the 441 patients, 161 (36.5%) had tumor recurrence during the median follow-up period of 61 months. During the postoperative period, 11 (2.5%) patients lost follow-up within 5 years after surgery. Patients with SRC were more likely to have tumor recurrence in the peritoneum than those without SRC, while non-SRC tumors metastasized more likely to the liver than SRC tumors. For early GC, there was no significant difference in the initial recurrence pattern between patients with and without SRC. For advanced GC with SRC, patients were more likely to develop peritoneal recurrence than those without SRC.

Among the 161 GC with tumor recurrence, 65 patients received 5-fluorouracil (5-FU)-based chemotherapy. Among the 65 patients receiving chemotherapy, one patient received radiofrequency ablation for single metastatic liver tumor; one patient received surgical excision for single metastatic lymph node above common hepatic artery and was disease-free for more than 10 years after that. The reasons for patients who had tumor recurrence but did not receive chemotherapy included no intention or not suitable for chemotherapy due to old age or poor general condition. Among the 161 GC with tumor recurrence, only 4 patients were alive until last follow-up.

### 2.3. The Correlation between Initial Recurrence Patterns and Genetic Alterations in Advanced GC

For advanced GC with SRC, patients with peritoneal recurrence had significantly more *PD-L1* expression (35.5% vs. 12.5%, *p =* 0.003), a trend of more *PIK3CA* amplifications (71.0% vs. 52.5%, *p =* 0.065), substantially fewer *ARID1A* mutations, but not statistically significant (0% vs. 9.2%, *p =* 0.080) and similar *PI3K/AKT* pathway mutations (9.7% vs. 13.3%, *p =* 0.584), *TP53* mutations (22.6% vs. 16.7%, *p =* 0.444), MSI-H tumors (3.2% vs. 2.5%, *p =* 0.822) compared with patients without peritoneal recurrence; patients with liver metastasis had no significant difference in *PD-L1* expression (8.3% vs. 18.0%, *p =* 0.395), *PIK3CA* amplifications (66.7% vs. 55.4%, *p =* 0.450), *PI3K/AKT* pathway mutations (25.0% vs. 11.5%, *p =* 0.176), *ARID1A* mutations (8.3% vs. 7.2%, *p =* 0.884), *TP53* mutations (8.3% vs. 18.7%, *p =* 0.368) and MSI-H tumors (0% vs. 2.9%, *p =* 0.551) compared with patients without liver metastasis.

For advanced GC without SRC, patients with peritoneal recurrence had similar *PD-L1* expression (41.7% vs. 26.1%, *p =* 0.109), *PIK3CA* amplifications (41.7% vs. 38.7%, *p =* 0.778), *PI3K/AKT* pathway mutations (25.0% vs. 19.7%, *p =* 0.542), *TP53* mutations (12.5% vs. 11.6%, *p =* 0.892), *ARID1A* mutations (8.3% vs. 15.6%, *p =* 0.345) and MSI-H tumors (12.5% vs. 12.6%, *p =* 0.993) compared with patients without peritoneal recurrence; patients with liver metastasis had significantly more *PD-L1* expression (46.9% vs. 24.6%, *p =* 0.009), more *PI3K/AKT* pathway mutations (34.4% vs. 17.9%, *p =* 0.032), more *TP53* mutations (25.0% vs. 9.4%, *p =* 0.011), similar *PIK3CA* amplifications (40.6% vs. 38.7%, *p =* 0.840) and *ARID1A* mutations (18.8% vs. 14.1%, *p =* 0.496) and significantly more MSI-H tumors (25.0% vs. 10.5%, *p =* 0.022) compared with patients without liver metastasis.

### 2.4. Survival Analysis

**Figure 1:**
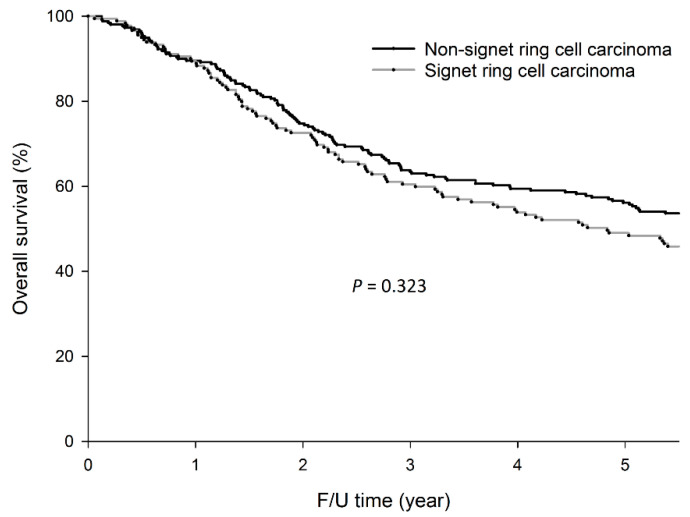
Five-year overall survival (OS) rates were not significantly different between patients with or without signet-ring cell carcinoma (SRC) (49.1% vs. 56.2%, *p =* 0.323).

For the 441 patients, the 5-year OS rates (49.1% vs. 56.2%, *p =* 0.323) were not significantly different between patients with and without SRC (Figure 1).

**Figure 2:**
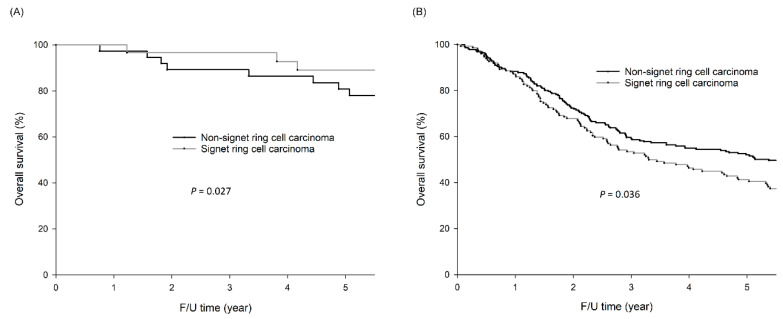
For early GC, the 5-year OS rates (89.1% vs. 80.8%, *p =* 0.027) were significantly better for patients with SRC than those without SRC. For advanced GC, the five-year OS rates (41.3% vs. 52.1%, *p =* 0.036) were significantly worse for patients with signet-ring cell carcinoma (SRC) than those without SRC. (**A**) OS curves of early GC patients with and without SRC; (**B**) OS curves of advanced GC patients with and without SRC.

As shown in Figure 2A, for early GC, the 5-year OS rates (89.1% vs. 80.8%, *p =* 0.027) were significantly better for patients with SRC than for those without SRC. For advanced GC (Figure 2B), the 5-year OS rates (41.3% vs. 52.1%, *p =* 0.036) were significantly worse for patients with SRC than for those without SRC.

Among the 181 SRC patients, 72 patients had tumor recurrence and 31 of them received 5-FU-based chemotherapy. Among the 72 SRC patients with tumor recurrence, there was no significant difference in the 5-year OS rates between patients receiving chemotherapy or not receiving chemotherapy (9.7% vs. 12.2%, *p =* 0.131).

Among the 260 non-SRC patients, 89 patients had tumor recurrence and 30 of them received 5-FU-based chemotherapy. Among the 89 non-SRC patients with tumor recurrence, the 5-year OS rates were significantly higher in patients receiving chemotherapy than those not receiving chemotherapy (20.0% vs. 11.9%, *p =* 0.017).

As shown in Table 5, univariate analysis showed that age, gender, tumor location, lymphovascular invasion, pathologic T and N categories and *PD-L1* expression were significantly associated with OS. The aforementioned seven factors were included in the multivariate analysis. Multivariate analysis with the Cox proportional hazard model showed that age and pathologic T and N categories were independent prognostic factors.

As shown in Table 6, for GC with SRC, univariate analysis showed that age, gender, tumor location, lymphovascular invasion, pathologic T and N categories and *ARID1A* mutation were significantly associated with OS. The aforementioned seven factors were included in the multivariate analysis. Multivariate analysis with the Cox proportional hazard model showed that age, gender, tumor location, lymphovascular invasion and pathologic T and N categories were independent prognostic factors.

As shown in Table S2, for GC without SRC, univariate analysis showed that age, gender, lymphovascular invasion and pathologic T and N categories were significantly associated with OS. The aforementioned five factors were included in the multivariate analysis. Multivariate analysis with the Cox proportional hazard model showed that age and pathologic N category were independent prognostic factors.

As shown in Table S3, for GC with tumor recurrence, univariate analysis showed that lymphovascular invasion and pathologic TNM stage were significantly associated with OS. The aforementioned two factors were included in the multivariate analysis. Multivariate analysis with the Cox proportional hazard model showed that lymphovascular invasion and pathologic TNM stage were independent prognostic factors.

## 3. Discussion

The present study showed that SRC patients were associated with a better prognosis in early GC and a worse prognosis in advanced GC compared with non-SRC patients. Furthermore, in early GC, SRC was associated with similar genetic alterations as non-SRC, whereas in advanced GC, SRC was associated with more *PIK3CA* amplifications and fewer MSI-H tumors than non-SRC. For advanced GC with SRC, patients who have peritoneal recurrence tended to express *PD-L1*, whereas for advanced GC without SRC, patients with liver metastases tended to have *PD-L1* expression, *PI3K/AKT* pathway mutations and *TP53* mutations.

Our results showed that in early GC, patients with SRC had a better prognosis than those without SRC, which was similar to the findings of other studies [6,7,8,9]. For early GC, although SRC tumors were associated with more unfavorable pathologic features such as more poorly differentiated and diffuse-type GC than non-SRC tumors, SRC tumors had better survival rates than non-SRC tumors. There was a trend in younger age and more HP infections (56.7% vs. 40.5%) in SRC tumors than in non-SRC tumors. For SRC tumors, the frequency of HP infections was significantly higher in early GC than in advanced GC (56.7% vs. 33.1%). It seems that HP infection may be involved in carcinogenesis in early GC with SRC. HP infections in GC were reported to be associated with a favorable prognosis [10,11]. In the present study, young age was also an independent favorable prognostic factor. Even in SRC, young age was associated with improved survival, even though younger patients were usually at a more advanced stage [12]. Consequently, young age and HP infection may explain why SRC tumors were associated with a better prognosis than non-SRC tumors in early GC.

For advanced GC, our results showed that SRC tumors were associated with worse survival than non-SRC tumors, which was similar to the findings of other reports [5,12]. In the present study, for advanced GC, univariate analysis showed that SRC tumors were associated with fewer MSI-H tumors, more *PIK3CA* amplifications and fewer *ARID1A* mutations than non-SRC tumors. *PIK3CA* amplifications were associated with a poor prognosis in GC [13]. *ARID1A* mutations were associated with MSI-H tumors [14]. Although the difference between SRC and non-SRC regarding the MSI status in GC has not yet been reported, a deficiency of mismatch repair genes was reported to be less frequent in SRC than in non-SRC in GC [15]. MSI-H tumors in GC were associated with a better prognosis than MSI-L/S tumors in GC [16]. As a result, genetic alterations may explain the worse prognosis of SRC than that of non-SRC tumors in advanced GC.

According to the TCGA database [17], GC was divided into four subtypes: (1) EBV-positive tumors, (2) microsatellite unstable tumors, (3) genomically stable tumors and (4) tumors with chromosomal instability. Among the four subtypes, EBV-positive tumor was associated with mutations in *PIK3CA* and *ARID1A* genes and elevated *PD-L1* expression. It was reported that GC patients with *ARID1A* mutations were associated with higher *PD-L1* expression than those without *ARID1A* mutations [18]. In GC with SRC or non-SRC, we hypothesized that EBV infection may have an impact on *PIK3CA* and *ARID1A* mutations, which may lead to elevated *PD-L1* expression. For better understanding, further in vivo and in vitro studies investigating the possible mechanism mentioned above is required.

A previous study [19] reported that patients with *PIK3CA* amplifications were associated with more peritoneal recurrence than those without *PIK3CA* amplifications in GC, which was similar to the findings of the present study (16.5% vs. 9.8%, *p =* 0.036). In the present study, SRC tumors were associated with more peritoneal recurrence than non-SRC tumors, especially in advanced GC, which was similar to the findings of a previous report [20]. It seems that *PIK3CA* amplifications in advanced GC with SRC play an important role in peritoneal recurrence. Based on our results, further investigation of the mechanism of *PIK3CA* in advanced GC with SRC may provide useful information for targeted therapy in the future.

*ARID1A*, a key component of the SWI/SNF chromatin remodeling complex, was reported to act as a tumor suppressor in various cancers [21]. It was reported that *ARID1A*-mutated GC was more common in MSI-H and EBV-associated GC [22]. Patients with *ARID1A* alterations had a longer recurrence-free survival and a better prognosis than those without *ARID1A* alterations [23]. In the present study, patients with *ARID1A* mutations had fewer peritoneal recurrence than those without *ARID1A* mutations (3.8% vs. 14.2%, *p =* 0.034). SRC tumors were associated with fewer MSI-H tumors and fewer *ARID1A* mutations than non-SRC tumors. Whether EBV infection has an impact on *ARID1A* alterations and MSI status in GC with SRC or non-SRC is unclear. For better understanding, further study is required to investigate the correlation between EBV infection, *ARID1A* alterations, other chromatin-modifying enzymes and MSI status in GC with SRC or non-SRC.

According to our results, it seems that chemotherapy may be beneficial for non-SRC GC with tumor recurrence, while no significant survival benefit of chemotherapy was observed for SRC GC with tumor recurrence. Because it is a retrospective study, selection bias exists, and the chemotherapy regimen used maybe not beneficial for SRC. In locally advanced, resectable gastric or gastro-esophageal junction adenocarcinoma, perioperative chemotherapy with fluorouracil plus leucovorin, oxaliplatin, and docetaxel was reported to improve overall survival compared with fluorouracil or capecitabine plus cisplatin and epirubicin, which was observed not only in intestinal-type tumors, but also in SRC tumors [24]. Consequently, in the view of multimodal treatment in GC, SRC tumors may need special considerations in the choice of best therapeutic option in GC.

It was reported that *PD-L1* expression was 25.7% in Krukenberg tumors which are metastatic SRCs from the stomach; *PD-L1* expression was associated with poor prognosis [25]. For advanced GC with SRC, CD^3+^ T cells were more infiltrated in *PD-L1*-positive tumors and further investigation of the cancer immunotherapy markers of SRC in GC may highlight targets for immunotherapy [26]. For our advanced GC patients, although *PD-L1* expression was less frequent in SRC than non-SRC, *PD-L1* expression (35.5% vs. 12.5%, *p =* 0.003) was significantly higher in patients with peritoneal recurrence than in patients without peritoneal recurrence. *PD-L1* expression may play an important role in peritoneal recurrence in advanced GC with SRC. For clinical practice, physicians should pay attention to the possibility of peritoneal recurrence in advanced GC with SRC and examinations for *PD-L1* are recommended for evaluation of the feasibility of immunotherapy.

In the present study, the definition of positive expression of *PD-L1* is a CPS of ≥ 1. In the KEYNOTE 059 study [27], anti-*PD-L1* antibody, pembrolizumab, demonstrated promising activity in advanced GC patients who had previously been treated with least a 2nd line therapy. Therefore, the Food and Drug Administration (FDA) grants approved the use of pembrolizumab for *PD-L1*-positive (CPS ≥ 1) GC. An anti-*PD-L1* antibody with 22C3 (Dako; Carpinteria, CA, USA) and CPS were adapted in the KEYNOTE 059 study, which was used in this study [28]. For advanced GC without SRC, our results showed that patients with liver metastasis had more *PD-L1* expression than patients without liver metastasis (46.9% vs. 24.6%, *p* < 0.001). We recommend checking *PD-L1* expression for this group of patients for the evaluation of immunotherapy.

There are some limitations in our study. It is retrospective and selection bias may exist. Although the present study enrolled a large population investigating the genetic alterations in GC with and without SRC and genetic alterations seemed to be associated with tumor recurrence patterns, more patients from different countries and of different races are required to validate our results. Because, the present study is a clinical study, we could only investigate the correlations between genetic alterations, clinicopathological features, recurrence patterns and patient prognosis. Further in vivo and in vitro studies are required to validate our results and the related mechanism, which may shed light on the future study and treatment of GC, including immunotherapy and targeted therapy.

## 4. Materials and Methods

### 4.1. Ethics Statement

All samples used in this study had been previously collected from the biobank of our hospital and were anonymized. All enrolled patients signed a consent form before sample collection in the biobank. All procedures implemented were in accordance with the ethical standards of the responsible committee on human experimentation (institutional and national) and with the Declaration of Helsinki of 1964 and its later versions. The ethics committees of our hospital approved this study (number: 2020–06–015CC).

### 4.2. Patients and Sample Collection

A total of 441 GC patients with adenocarcinoma who underwent curative surgery between January 2005 and December 2013 were enrolled in this study. Patients with hereditary diffuse type GC were excluded. In the WHO classification [2] of GC histological classification, GC was classified as papillary, tubular, mucinous, signet-ring cell and undifferentiated adenocarcinoma. In the present study, SRC was defined according to the classification of the WHO [2], for which malignant tumor cells with prominent mucin in the cytoplasm and eccentric crescent-shaped nuclei were present in more than 50% of the GC tumor; non-SRC GC was defined as GC other than SRC, including papillary, tubular, mucinous and undifferentiated adenocarcinoma. According to the definition of the Japanese classification of gastric carcinoma, early GC was defined as pathologic T1 tumor irrespective of lymph node metastasis, while advanced GC was defined as pathologic T2–T4 tumor [29].

Normal and tumor tissues were immediately frozen in liquid nitrogen and stored in the biobank at our institution. The gross features of the pathologic specimens were evaluated according to the tumor location, tumor size and Borrmann’s classification. The microscopic features of histology, pathology and cell differentiation were analyzed according to the cell grade of tumor differentiation, the stromal reaction type (medullary, intermediate and scirrhous types), Ming’s histological classification (expanding or infiltrating type) and lymphovascular invasion patterns. The pathologic staging of GC was defined according to the 8th American Joint Committee on Cancer (AJCC)/Union for International Cancer Control (UICC) TNM classification of malignant tumors [30]. All surgical specimens were examined by experienced pathologists. All surgeries were performed by surgeons who specialized in GC. The data were retrospectively collected and recorded with a computer, and the conditions of the patients throughout the follow-up period were regularly updated.

Prior to surgery, all patients underwent chest X-ray, tumor markers (e.g., carcinoembryonic antigen and carbohydrate antigen 19–9) and a computerized tomography (CT) scan of abdomen. If lung nodules or tumors were suspected by chest X-ray or CT scan of abdomen, chest CT scan was arranged. Whole body bone scan was performed for patients with the presentation of bone pain. Distant metastasis was diagnosed by biopsies or by imaging studies when biopsies were not obtained. A total or distal subtotal gastrectomy was performed according to the tumor location. For early GC, at least limited lymph node dissection (D1+) was performed, whereas extended lymph node dissection (D2) was performed for advanced GC [29].

### 4.3. Follow-Up

Postoperative follow-up assessments were performed as per local practice guidelines: every 3 months for the first 3 years and every 6 months thereafter until the patient’s death. The follow-up procedures included physical examinations, blood tests including tumor markers (e.g., carcinoembryonic antigen and carbohydrate antigen 19–9), liver function tests, chest films, abdominal sonography and computerized tomography scans. Tumor recurrence was diagnosed by biopsies or by imaging studies when biopsies were not obtained. Tumor recurrence in the hepatoduodenal ligament, celiac axis or peripancreatic region was considered locoregional recurrence. We defined remote lymphatic metastasis (in the para-aortic, Virchow’s and inguinal nodes) and pulmonary lymphangitic spread as distant lymphatic recurrence. Tumor recurrence was classified as locoregional, hematogenous, distant lymphatic or peritoneal. Patients with tumor recurrence were eligible to receive 5-FU-based chemotherapy. None of the patients enrolled received neoadjuvant chemotherapy, targeted therapy or sophisticated regimens. Since 2008, adjuvant therapy (such as S-1) was prescribed for Stage II or Stage III disease after curative surgery at our hospital due to its demonstrated survival benefit [31]. Among the 441 patients, 8 SRC patients and 4 non-SRC patients received adjuvant chemotherapy. Before December 2016, the cost of S-1 was not covered by our National Health Insurance and patients needed to pay for S-1 adjuvant chemotherapy by themselves, which caused a relatively low rate of adjuvant chemotherapy in the present study.

### 4.4. DNA Extraction

As a previous report [32], DNA was extracted from fresh frozen or formalin-fixed paraffin-embedded tissue specimens using the QIAamp DNA Tissue Kit.

### 4.5. Identification of Helicobacter pylori (HP) Infection

For identification of HP infection, we examined both tumor and nontumor tissues. As described in our previous study [33], the polymerase chain reaction (PCR) method was used to identify HP infection.

### 4.6. PIK3CA Amplification

The copy number of the *PIK3CA* gene was analyzed by quantitative real-time polymerase chain reaction and the primer sequences of the long interspersed nuclear element-1 (LINE1 element) were used as an internal reference target. The method of identifying *PIK3CA* amplification was the same as that described in a previous study [17]. The relative copy number in each sample was determined by comparing the ratio of *PIK3CA* gene to the LINE1 element. Amplification of the *PIK3CA* gene was defined as a copy number of ≥ 3 with *p*-value of < 0.05.

### 4.7. MassARRAY-Based Mutation Characterization

A sensitive MassARRAY system (Agena, San Diego, CA, USA) was used to identify 76 mutation hotspots in nine common GC-related genes as shown in Table S1 (including *PIK3CA*, *PTEN*, *AKT1*, *AKT2*, *AKT3*, *ARID1A*, *TP53*, *BRAF* and *KRAS*), which were reported in previous studies [17,32,34]. The *PI3K/AKT* pathway genetic mutation included five genes: *PIK3CA*, *PTEN*, *AKT1*, *AKT2* and *AKT3*.

### 4.8. Microsatellite Instability Analysis

As mentioned in a previous study [16], five reference microsatellite markers, D5S345, D2S123, D17S250, BAT25 and BAT26, were used to determine MSI. MSI-H was defined as samples with ≥ 2 loci of instability with 5 markers; MSI-low/stable (MSI-L/S) was defined as samples with one MSI or without MSI.

### 4.9. Immunohistochemical (IHC) Staining for PD-L1

**Figure 3:**
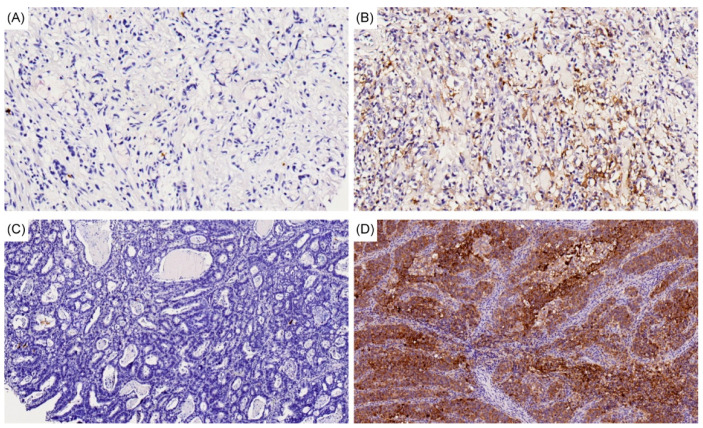
PD-L1 IHC staining results with 200× magnification in each histological type of GC are shown as follows: (**A**) negative staining in GC with SRC; (**B**) positive staining in GC with SRC; (**C**) negative staining in GC with non-SRC; (**D**) positive staining in GC with non-SRC.

For *PD-L1* expression, IHC staining was performed using the *PD-L1* IHC 22C3 pharmDx kit on the Dako ASL48 platform [35]. The combined positive score (CPS) consisted of the number of *PD-L1*-stained cells, including tumor cells, lymphocytes and macrophages, relative to the number of all viable tumor cells. The CPS was calculated and a CPS score of ≥ 1 was defined as positive expression of *PD-L1*. Sections with both negative and positive control were provided by Dako pharmDx and the quality control was performed in each batch of stains. For SRC tumors, Figure 3A shows the negative staining of *PD-L1* and Figure 3B demonstrates the positive staining of *PD-L1*. For non-SRC tumors, Figure 3C shows the negative staining of *PD-L1* and Figure 3D demonstrates the positive staining of *PD-L1*.

### 4.10. Statistical Analysis

Statistical analyses were performed using IBM SPSS Statistics 25.0 (Armonk, New York, NY, USA: IBM Corp). The categorical data were compared using a χ^2^ test with Yates correction or Fisher’s exact test. Corrections of data were performed using multiple testing in logistic regression model. Overall survival (OS) was measured from the operation date to the date of death or the final follow-up visit. The distributions of OS were estimated using the Kaplan–Meier method. Univariate analysis of the covariates (prognostic factors) of OS was performed first. The covariates with *p*-value < 0.05 were selected for the entry of Cox proportional hazards model. Multivariate analysis using Cox proportional hazards model with likelihood ratio (forward stepwise) test for several steps of iteration was performed with *p*-value < 0.05 as entry and *p*-value > 0.1 as removal. A *p*-value of less than 0.05 was considered statistically significant.

## 5. Conclusions

Our results demonstrated that in early GC, SRC was associated with similar genetic mutations and better survival rates than those without SRC. Advanced GC patients with SRC who developed peritoneal recurrence tended to have more *PD-L1* expression, while advanced GC patients without SRC who had liver metastasis are likely to have *PI3K/AKT* pathway mutations, *TP53* mutations and *PD-L1* expression. Consequently, immunotherapy and targeted therapy may be beneficial for these patients.

## Figures and Tables

**Table 1:** Clinical profile of gastric cancer (GC) patients with and without signet-ring cell carcinoma (SRC).

Variables	Univariate Analysis			Multiple Testing Correction Logistic Regression		
	Non-SRC *n* = 260 *n* (%)	SRC *n* = 181 *n* (%)	*p*-Value	Odds Ratio	Confidence Interval	*p*-Value
Age			**<0.001**			0.099
<65 years	82 (31.5)	96 (53.0)		1.00		
≥65 years	178 (68.5)	85 (47.0)		0.65	0.394–1.083	
Gender			**<0.001**			**0.013**
Male	203 (78.1)	108 (59.7)		1.00		
Female	57 (21.9)	73 (40.3)		2.02	1.163–3.493	
Tumor size			0.165			
<5cm	109 (41.9)	64 (35.4)				
≥5 cm	151 (58.1)	117 (64.6)				
Tumor location			**<0.001**			0.311
Upper stomach	55 (21.2)	25 (13.8)		1.00		
Middle stomach	70 (26.9)	76 (42.0)		2.40	1.187–4.843	
Lower stomach	133 (51.2)	71 (39.2)		1.07	0.543–2.097	
Whole stomach				4.36	0.681–27.877	
Stromal reaction type			**<0.001**			**<0.001**
Medullary	47 (18.1)	18 (9.9)		1.00		
Intermediate	181 (69.6)	57 (31.5)		0.54	0.237–1.216	
Scirrhous	32 (12.3)	106 (58.6)		4.28	1.748–10.500	
Ming’s classification			**<0.001**			**<0.001**
Expanding	99 (38.1)	14 (7.7)		1.00		
Infiltrating	161 (61.9)	167 (92.3)		3.96	1.938–8.105	
Extent of lymphadenectomy			0.751			
Limited (D1+)	57 (21.9)	42 (23.2)				
Extended (D2)	203 (78.1)	139 (76.8)				
Lymphovascular invasion	187 (71.9)	124 (68.5)	0.439			
Lymphoid stroma	38 (14.6)	25 (13.8)	0.813			
MSI status			**0.001**			**0.008**
MSI-L/S	229 (88.1)	175 (96.7)		1.00		
MSI-H	31 (11.9)	6 (3.3)		0.23	0.076–0.684	
HP infection	90 (34.6)	67 (37.0)	0.604			
*PIK3CA* amplification	106 (40.8)	100 (55.2)	**0.003**	1.71	1.025–2.855	**0.040**
*PD-L1* expression	68 (26.2)	28 (15.5)	**0.007**	0.68	0.352–1.299	0.240
Genetic mutation						
*PI3K/AKT* pathway	49 (18.9)	21 (11.6)	**0.039**	0.82	0.389–1.708	0.589
*ARID1A*	37 (14.2)	16 (8.8)	0.087			
*TP53*	32 (12.3)	30 (16.6)	0.205			
*KRAS*	9 (3.5)	1 (0.6)				
*BRAF*	3 (1.2)	0				
Pathologic TNM Stage			**0.022**			0.774
I	60 (23.1)	28 (15.5)		1.00		
II	80 (30.8)	46 (25.4)		1.13	0.516–2.456	
III	120 (46.2)	107 (59.1)		1.29	0.607–2.741	

GC—gastric cancer; SRC—signet-ring cell; MSI—microsatellite instability; MSI-H—microsatellite instability-high; MSI-L/S—microsatellite instability-low/stable; HP—*Helicobacter pylori*; EBV—Epstein–Barr virus; bold—statistically significant.

**Table 2:** Clinical profile in early GC patients with and without SRC.

Variables	Univariate Analysis			Multiple Testing Correction Logistic Regression		
	Non-SRC *n* = 37 *n* (%)	SRC *n* = 30 *n* (%)	*p* Value	Odds Ratio	Confidence Interval	*p* Value
Age			0.064			
<65 years	15 (40.5)	19 (63.3)				
≥65 years	22 (59.5)	11 (36.7)				
Gender			**0.016**			0.130
Male	22 (59.5)	9 (30.0)		1.00		
Female	15 (40.5)	21 (70.0)		2.69	0.747–9.681	
Tumor size			0.981			
<5 cm	26 (70.3)	21 (70.0)				
≥5 cm	11 (29.7)	9 (30.0)				
Tumor location			**0.024**			**0.036**
Upper stomach	11 (29.7)	2 (6.7)		1.00		
Middle stomach	14 (37.8)	20 (66.7)		10.37	1.456–73.868	
Lower stomach	12 (32.4)	8 (26.7)		2.64	0.360–19.366	
Stromal reaction type			0.053			
Medullary	19 (51.4)	16 (53.3)				
Intermediate	16 (43.2)	7 (23.3)				
Scirrhous	2 (5.4)	7 (23.3)				
Ming’s classification			**<0.001**			**<0.001**
Expanding	25 (67.6)	4 (13.3)		1.00		
Infiltrating	12 (32.4)	26 (86.7)		13.93	3.319–58.439	
Extent of lymphadenectomy			0.557			
Limited (D1+)	11 (29.7)	7 (23.3)				
Extended (D2)	26 (70.3)	23 (76.7)				
Lymphovascular invasion	7 (18.9)	4 (13.3)	0.539			
Lymphoid stroma	5 (13.5)	3 (10.0)	0.659			
MSI status			0.823			
MSI-L/S	34 (91.9)	28 (93.3)				
MSI-H	3 (8.1)	2 (6.7)				
HP infection	15 (40.5)	17 (56.7)	0.189			
*PIK3CA* amplification	19 (51.4)	15 (50.0)	0.912			
*PD-L1* expression	6 (16.2)	2 (6.7)	0.231			
Genetic mutation						
*PI3K/AKT* pathway	4 (10.8)	2 (6.7)	0.555			
*ARID1A*	4 (10.8)	5 (16.7)	0.485			
*TP53*	6 (16.2)	3 (10.0)	0.458			
*KRAS*	1 (2.7)	0				
*BRAF*	0	0				
Pathologic TNM Stage			0.129			
I	36 (97.3)	25 (83.3)				
II	1 (2.7)	4 (13.3)				
III	0	1 (3.3)				

GC—gastric cancer; SRC—signet-ring cell; MSI—microsatellite instability; MSI-H—microsatellite instability-high; MSI-L/S—microsatellite instability-low/stable; HP—*Helicobacter pylori*; EBV—Epstein–Barr virus; bold—statistically significant.

**Table 3:** Clinical profile in advanced GC patients with and without SRC.

Variables	Univariate Analysis			Multiple Testing Correction Logistic Regression		
	Non-SRC *n* = 223 *n* (%)	SRC *n* = 151 *n* (%)	*p* Value	Odds Ratio	Confidence Interval	*p* Value
Age			**<0.001**			0.124
<65 years	67 (30.0)	77 (51.0)		1.00		
≥65 years	156 (70.0)	74 (49.0)		0.65	0.371–1.128	
Gender			**0.001**			0.090
Male	181 (81.2)	99 (65.6)		1.00		
Female	42 (18.8)	52 (34.4)		1.73	0.917–3.271	
Tumor size			0.079			
<5 cm	83 (37.2)	43 (28.5)				
≥5 cm	140 (62.8)	108 (71.5)				
Tumor location			**<0.001**			0.070
Upper stomach	44 (19.7)	23 (15.2)		1.00		
Middle stomach	56 (25.1)	56 (37.1)		1.85	0.843–4.060	
Lower stomach	121 (54.3)	63 (41.7)		0.97	0.462–2.014	
Whole stomach	2 (0.9)	9 (6.0)		5.10	0.717–36.346	
Stromal reaction type			**<0.001**			**<0.001**
Medullary	28 (12.6)	2 (1.3)		1.00		
Intermediate	165 (74.0)	50 (33.1)		2.37	0.475–11.779	
Scirrhous	30 (13.5)	99 (65.6)		21.47	4.150–111.092	
Ming’s classification			**<0.001**			0.066
Expanding	74 (33.2)	10 (6.6)		1.00		
Infiltrating	149 (66.8)	141 (93.4)		2.19	0.949–5.066	
Extent of lymphadenectomy			0.557			
Limited (D1+)	46 (20.6)	35 (23.2)				
Extended (D2)	177 (79.4)	116 (76.8)				
Lymphovascular invasion	180 (80.7)	120 (79.5)	0.766			
Lymphoid stroma	33 (14.8)	22 (14.6)	0.951			
MSI status			**0.001**			**0.012**
MSI-L/S	195 (87.4)	147 (97.4)		1.00		
MSI-H	28 (12.6)	4 (2.6)		0.19	0.024–0.696	
HP infection	75 (33.6)	50 (33.1)	0.917			
*PIK3CA* amplification	87 (39.0)	85 (56.3)	**0.001**	2.00	1.165–3.427	**0.012**
*PD-L1* expression	62 (27.8)	26 (17.2)	**0.018**	0.72	0.359–1.438	0.350
Genetic mutation						
*PI3K/AKT* pathway	45 (20.3)	19 (12.6)	0.053			
*ARID1A*	33 (14.8)	11 (7.3)	**0.027**	0.47	0.191–1.162	0.102
*TP53*	26 (11.7)	27 (17.9)	0.091			
*KRAS*	8 (3.6)	1 (0.7)				
*BRAF*	3 (1.3)	0				
Pathologic TNM Stage			**<0.001**			0.416
I	24 (10.8)	3 (2.0)		1.00		
II	79 (35.4)	42 (27.8)		1.75	0.447–6.867	
III	120 (53.8)	106 (70.2)		2.24	0.585–8.545	

GC—gastric cancer; SRC—signet-ring cell; MSI—microsatellite instability; MSI-H—microsatellite instability-high; MSI-L/S—microsatellite instability-low/stable; HP—*Helicobacter pylori*; EBV—Epstein–Barr virus; bold—statistically significant.

**Table 4:** The initial recurrence pattern in GC patients with and without SRC after curative surgery.

Initial Recurrence Pattern	All GC Patients				Early GC Patients			Advanced GC Patients		
	All Recurrence *n*	Non-SRC *n* = 260 *n* (%)	SRC *n* = 181 *n* (%)	*p* Value	Non-SRC *n* = 37 *n* (%)	SRC *n* = 30 *n* (%)	*p*-Value	Non-SRC *n* = 223 *n* (%)	SRC *n* = 151 *n* (%)	*p* Value
Total patients with recurrence	161	89 (34.2)	72 (39.8)	0.234	4 (10.8)	4 (13.3)	0.752	85 (38.1)	68 (45.0)	0.182
Locoregional recurrence	61	38 (14.6)	23 (12.7)	0.568	1 (2.7)	1 (3.3)	0.880	37 (16.6)	22 (14.6)	0.599
Distant metastasis	126	71 (27.3)	55 (30.4)	0.481	5 (13.5)	2 (6.7)	0.362	66 (29.6)	53 (35.1)	0.262
Peritoneal dissemination	57	25 (9.6)	32 (17.7)	**0.013**	1 (2.7)	1 (3.3)	0.880	24 (10.8)	31 (20.5)	**0.009**
Hematogenous metastasis	66	42 (16.2)	24 (13.3)	0.402	4 (10.8)	1 (3.3)	0.247	38 (17.0)	23 (15.2)	0.642
Liver	45	33 (12.7)	12 (6.6)	**0.039**	1 (2.7)	0		32 (14.3)	12 (7.9)	0.059
Lung	12	4 (1.5)	8 (4.4)		1 (2.7)	1 (3.3)		3 (1.3)	7 (4.6)	
Bone	11	6 (2.3)	5 (2.8)		1 (2.7)	1 (3.3)		5 (2.2)	4 (2.6)	
Brain	1	1 (0.4)	0		0	0		1 (0.4)	0	
Adrenal	3	1 (0.4)	2 (1.1)		1 (2.7)	0		0	2 (1.3)	
Skin	4	2 (0.8)	2 (1.1)		1 (2.7)	0		1 (0.4)	2 (1.3)	
Distant lymphatic recurrence	36	19 (7.3)	17 (9.4)	0.432	1 (2.7)	0	0.364	18 (8.1)	17 (11.3)	0.299
Virchow’s node	6	3 (1.2)	3 (1.7)		0	0		3 (1.3)	3 (2.0)	
Lymphangitis carcinomatosis	1	0	1 (0.6)		0	0		0	1 (0.7)	
Para-aortic lymph node	31	17 (6.5)	14 (7.7)		1 (2.7)	0		16 (7.2)	14 (9.3)	

Some patients had more than one recurrence pattern; bold—statistically significant Some patients had more than one recurrence pattern; GC—gastric cancer; SRC—signet-ring cell; bold—statistically significant.

**Table 5:** Univariate and multivariate analyses of factors affecting OS of all the enrolled GC patients after curative surgery by the Cox proportional hazards model.

Factors	Univariate Analysis			Multivariate Analysis		
	Hazard Ratio	95% Confidence Interval	*p*-Value	Hazard Ratio	95% Confidence Interval	*p*-Value
Age (y/o)			**<0.001**			**<0.001**
<65	1.00			1.00		
≥65	1.74	1.347–2.247		1.90	1.464–2.467	
Gender			**<0.001**			
Male	1.00					
Female	0.59	0.442–0.780				
Tumor location			**0.007**			
Upper third stomach	1.00					
Middle third stomach	0.72	0.508–1.102				
Lower third stomach	0.85	0.623–1.169				
Whole stomach	2.26	1.113–4.581				
Lymphovascular invasion			**<0.001**			
Absent	1.00					
Present	2.41	1.783–3.251				
Pathologic T category			**<0.001**			**<0.001**
T1	1.00			1.00		
T2	1.64	0.964–2.780		1.20	0.689–2.079	
T3	2.65	1.678–4.188		1.57	0.959–2.583	
T4	4.39	2.810–6.859		2.49	1.531–4.056	
Pathologic N category			**<0.001**			**<0.001**
N0	1.00			1.00		
N1	0.99	0.647–1.501		0.95	0.614–1.465	
N2	2.03	1.455–2.843		1.63	1.138–2.323	
N3	5.13	3.711–7.099		4.41	3.080–6.301	
MSI status			0.627			
MSI-L/S	1.00					
MSI-H	0.63	0.725–1.704				
*PIK3CA* amplification			0.068			
Absent	1.00					
Present	1.25	0.984–1.579				
*PD-L1* expression			**0.044**			
Negative	1.00					
Positive	1.32	1.007–1.737				
*PI3K/AKT* pathway mutation			0.945			
Absent	1.00					
Present	0.95	0.689–1.298				
*TP53* mutation			0.144			
Absent	1.00					
Present	1.27	0.921–1.756				
*ARID1A* mutation			0.237			
Absent	1.00					
Present	0.80	0.557–1.156				
Cancer cell type			0.236			
Non-SRC	1.00					
SRC	1.16	0.910–1.467				

OS—overall survival; MSI—microsatellite instability; MSI-H—microsatellite instability-high; MSI-L/S—microsatellite instability-low/stable; SRC—signet-ring cell; bold—statistically significant.

**Table 6:** Univariate and multivariate analyses of factors affecting OS of GC patients with SRC after curative surgery by the Cox proportional hazards model.

Factors	Univariate Analysis			Multivariate Analysis		
	Hazard Ratio	95% Confidence Interval	*p*-Value	Hazard Ratio	95% Confidence Interval	*p*-Value
Age (y/o)			**0.009**			**0.020**
<65	1.00			1.00		
≥65	1.63	1.132–2.345		1.63	1.082–2.468	
Gender			**0.001**			
Male	1.00					
Female	0.51	0.344–0.759				
Tumor location			**0.014**			**0.008**
Upper third stomach	1.00			1.00		
Middle third stomach	0.52	0.301–0.889		0.50	0.279–0.882	
Lower third stomach	0.66	0.387–1.120		0.37	0.204–0.664	
Whole stomach	1.55	0.647–3.686		0.66	0.254–1.701	
Lymphovascular invasion			**<0.001**			**0.044**
Absent	1.00			1.00		
Present	3.89	2.377–6.373		1.90	1.017–3.536	
Pathologic T category			**<0.001**			**0.016**
T1	1.00			1.00		
T2	3.38	1.227–9.299		1.52	0.505–4.557	
T3	5.44	2.314–12.779		1.78	0.684–4.611	
T4	8.95	3.847–20.827		3.20	1.191–8.618	
Pathologic N category			**<0.001**			**<0.001**
N0	1.00			1.00		
N1	1.28	0.632–2.571		0.91	0.424–1.935	
N2	2.76	1.547–4.913		1.90	1.030–3.522	
N3	5.34	3.078–9.271		4.26	2.265–7.994	
MSI status			0.439			
MSI-L/S	1.00					
MSI-H	0.64	0.201–2.004				
*PIK3CA* amplification			0.172			
Absent	1.00					
Present	1.29	0.894–1.870				
*PD-L1* expression			0.177			
Negative	1.00					
Positive	1.39	0.863–2.222				
*PI3K/AKT* pathway mutation			0.823			
Absent	1.00					
Present	0.94	0.547–1.617				
*TP53* mutation			0.488			
Absent	1.00					
Present	1.18	0.741–1.876				
*ARID1A* mutation			**0.041**			
Absent	1.00					
Present	0.45	0.209–0.968				

OS—overall survival: MSI—microsatellite instability; MSI-H—microsatellite instability-high; MSI-L/S—microsatellite instability-low/stable; SRC—signet-ring cell; bold—statistically significant.

## Supplementary Materials

The following are available online at https://www.mdpi.com/2072-6694/12/8/2318/s1, Table S1. The 76 target mutations in 9 genes in GC, Table S2. Univariate and multivariate analyses of factors affecting OS of GC patients without SRC after curative surgery by the Cox proportional hazards model, Table S3. Univariate and multivariate analyses of factors affecting OS of GC patients with tumor recurrence after curative surgery by the Cox proportional hazards model.

## Abbreviations

AJCC	American Joint Committee on Cancer
CPS	combined positive score
DFS	disease-free survival
EBV	Epstein–Barr virus
GC	gastric cancer
HP	*Helicobacter pylori*
IHC	immunohistochemical
MSI	microsatellite instability
MSI-H	microsatellite instability—high
MSI-L/S	microsatellite instability—low/stable
OS	overall survival
PCR	polymerase chain reaction
PD-L1	programmed death-ligand 1
UICC	Union for International Cancer Control
SRC	Signet-ring cell
TNM	tumor, node, metastasis
WHO	World Health Organization
