# Supplementary material for: The Clinicopathological Characteristics And Genetic Alterations of Signet-ring Cell Carcinoma in Gastric Cancer
Source: Cancers (Basel). 2020 Aug 17;12(8):2318. doi: 10.3390/cancers12082318 (PMC7463705; doi:10.3390/cancers12082318)
Supplement: Supplementary file 1 [file cancers-12-02318-s001.pdf]

cancers

MDPI

Article

The Clinicopathological Characteristics And Genetic Alterations of Signet-ring Cell Carcinoma in Gastric Cancer

Kuo-Hung Huang, Ming-Huang Chen, Wen-Liang Fang, Chien-Hsing Lin, Yee Chao, Su-Shun Lo, Anna Fen-Yau Li, Chew-Wun Wu and Yi-Ming Shyr

Supplementary Materials

Table S1. The 76 target mutations in 9 genes in GC.

Gene	cDNA change	Amino acid mutation
AKT1	c.49G>A	p.E17K
AKT2	c.1063C>T	p.H355Y
	c.904A>G	p.S302G
	c.1112G>A	p.R371H
AKT3	c.514A>C	p.K172Q
	c.496C>T	p.R166*
	c.49G>A	p.E17K
	c.1080G>A	p.M360I
	c.1100G>A	p.R367Q
	c.1192G>T	p.E398*
PIK3CA	c.263G>A	p.R88Q
	c.1035T>A	p.N345K
	c.1258T>C	p.C420R
	c.1616C>G	p.P539R
	c.1624G>A	p.E542K
	c.1633G>A	p.E545K
	c.2102A>C	p.H701P
	c.3140A>T	p.H1047L
	c.3140A>G	p.H1047R
	c.3139C>T	p.H1047Y
	c.1634A>C	p.E545A
	c.3204_3205insA	p.N1068fs*4
	c.1634A>G	p.E545G
	c.3062A>G	p.Y1021C
	c.331A>G	p.K111E
PTEN	c.388C>T	p.R130*
	c.388C>G	p.R130G
	c.389G>A	p.R130Q
	c.517C>T	p.R173C
	c.518G>A	p.R173H
	c.697C>T	p.R233*
	c.1003C>T	p.R335*
	c.968_969insA	p.N323fs*2
	c.968delA	p.N323fs*21
	c.800delA	p.K267fs*9
	c.951_954delACTT	p.V317fs*3

	c.741_742insA	p.P248fs*5
	c.389delG	p.R130fs*4
	c.16_17delAA	p.K6fs*4
ARID1A	c.3826C>T	p.R1276*
	c.5965C>T	p.R1989*
	c.5542delG	p.D1850fs*33
	c.5161C>T	p.R1721*
	c.2077C>T	p.R693*
	c.6415delC	p.F2141fs*59
	c.5164C>T	p.R1722*
	c.2830C>T	p.Q944*
	c.4003C>T	p.R1335*
	c.5548_5549insG	p.D1850fs*4
	c.1242A>G	p.G414G
	c.6259G>A	p.G2087R
	c.4582C>T	p.R1528*
TP53	c.524G>A	p.R175H
	c.637C>T	p.R213*
	c.659A>G	p.Y220C
	c.672+2T>G	p.RFX6
	c.733G>A	p.G245S
	c.742C>T	p.R248W
	c.743G>A	p.R248Q
	c.743G>T	p.R248L
	c.747G>T	p.R249S
	c.817C>T	p.R273C
	c.818G>A	p.R273H
	c.818G>T	p.R273L
	c.844C>T	p.R282W
	c.844C>G	p.R282G
	c.916C>T	p.R306*
BRAF	c.1799T>A	p.V600E
KRAS	KRAS c.35G>A	p.G12D
	KRAS c.35G>C	p.G12A
	KRAS c.35G>T	p.G12V
	KRAS c.38G>A	p.G13D
	KRAS c.175G>A	p.A59T
	KRAS c.183A>C	p.Q61H
	KRAS c.436G>A	p.A146T
	KRAS c.437C>T	p.A146V

Table S2. Univariate and multivariate analyses of factors affecting OS of GC patients without SRC after curative surgery by the Cox proportional hazards model.

	Univariate analysis			Multivariate analysis		
Factors	Hazard ratio	95% Confidence interval	*P* value	Hazard ratio	95% Confidence interval	*P* value
Age (y/o)			**<0.001**			**<0.001**
<65	1.00			1.00		
≧65	2.08	1.418-3.057		2.21	1.488-3.287	
Gender			**0.022**			
Male	1.00					
Female	1.63	1.073-2.488				

Tumor location			0.236			
Upper third stomach	1.00					
Middle third stomach	0.81	0.507-1.278				
Lower third stomach	0.94	0.638-1.395				
Whole stomach	3.38	0.809-14.146				
Lymphovascular invasion			**0.005**			
Absent	1.00					
Present	1.73	1.185-2.537				
Pathological T category			**<0.001**			
T1	1.00					
T2	1.11	0.598-2.073				
T3	1.68	0.967-2.931				
T4	2.85	1.674-4.857				
Pathological N category			**<0.001**			**<0.001**
N0	1.00			1.00		
N1	0.85	0.499-1.454		0.87	0.498-1.533	
N2	1.72	1.129-2.614		1.53	0.956-2.445	
N3	5.44	3.574-8.273		4.98	3.057-8.105	
MSI status			0.233			
MSI-L/S	1.00					
MSI-H	1.33	0.832-2.128				
PIK3CA amplification			0.303			
Absent	1.00					
Present	1.18	0.860-1.622				
PD-L1 expression			0.078			
Negative	1.00					
Positive	1.36	0.967-1.906				
PI3K/AKT pathway mutation			0.970			
Absent	1.00					
Present	0.97	0.654-1.437				
TP53 mutation			0.191			
Absent	1.00					
Present	1.35	0.860-2.121				
ARID1A mutation			0.840			
Absent	1.00					
Present	1.05	0.685-1.593				

OS: overall survival; SRC: signet-ring cell carcinoma; MSI: microsatellite instability; MSI-L/S: microsatellite instability-low/stable; MSI-H: microsatellite instability-high; bold: statistically significant.

Table S3. Univariate and multivariate analyses of factors affecting OS of GC patients with tumor recurrence after curative surgery by the Cox proportional hazards model.

	Univariate analysis			Multivariate analysis		
Factors	Hazard ratio	95% Confidence interval	*P* value	Hazard ratio	95% Confidence interval	*P* value
Age (y/o)			0.197			
<65	1.00					
≧65	1.24	0.896-1.706				
Gender			0.126			
Male	1.00					
Female	1.33	0.923-1.909				
Tumor location			0.222			
Upper third stomach	1.00					
Middle third stomach	0.71	0.445-1.134				
Lower third stomach	0.91	0.602-1.375				

Whole stomach	1.80	0.633-5.123				
Lymphovascular invasion			**0.002**			**0.037**
Absent	1.00			1.00		
Present	2.13	1.318-3.430		1.70	1.033-2.810	
Pathological TNM stage			**0.004**			**0.016**
I	1.00			1.00		
II	2.36	0.979-5.702		2.08	0.855-5.069	
III	3.49	1.515-8.035		2.76	1.167-6.527	
MSI status			0.696			
MSI-L/S	1.00					
MSI-H	1.11	0.651-1.900				
PIK3CA amplification			0.922			
Absent	1.00					
Present	1.02	0.742-1.391				
PD-L1 expression			0.695			
Negative	1.00					
Positive	0.90	0.533-1.521				
PI3K/AKT pathway mutation			0.520			
Absent	1.00					
Present	0.88	0.582-1.315				
TP53 mutation			0.123			
Absent	1.00					
Present	1.42	0.909-2.222				
ARID1A mutation			0.709			
Absent	1.00					
Present	0.71	0.447-1.124				
Cancer cell type			0.374			
Non-SRC	1.00					
SRC	1.16	0.841-1.586				

OS: overall survival; MSI: microsatellite instability; MSI-H: microsatellite instability-high; MSI-L/S: microsatellite instability-low/stable; SRC: signet-ring cell carcinoma; bold: statistically significant.
